# MiR-6839-5p inhibits cell proliferation, migration and invasion; a possible correlation with the suppressing VEGFA expression in human chondrosarcoma cells

**DOI:** 10.1007/s12672-024-01038-5

**Published:** 2024-05-18

**Authors:** Fusheng Li, Jia Xu, Yue Zhu

**Affiliations:** 1 Department of Orthopaedics, The First Affiliated Hospital of China Medical University, 155 Nan Jing Bei Street, Shenyang, 110001 People’s Republic of China; 2 Department of Orthopaedics Oncology, The People’s Hospital of Liaoning Province, Shenyang, 110016 People’s Republic of China; 3 Department of Medical Microbiology, Key Laboratory of Environmental Pollution and Microecology of Liaoning Province, Shenyang Medical College, Shenyang, 110034 People’s Republic of China

**Keywords:** Chondrosarcoma, miR-6839-5p, BCL-2, VEGFA, Apoptosis, Proliferation

## Abstract

MicroRNAs play an important role in the proliferation, invasion, and metastasis of malignancy. In previous studies (detailed in our previous paper), the expression of miR-6839-5p was significantly increased in SW1353 cells after ^125^I seed 6 Gy irradiation, which indicated miR-6839-5p may play a tumor suppression function in chondrosarcoma cells. This study aimed to identify the effects of miR-6839-5p on the human chondrosarcoma cells, and investigate the potential target genes of miR-6839-5p. Firstly, chondrosarcoma cells (SW1353 and CAL78) were transfected with hsa-miR-6839-5p specific mimic. Secondly, Cell viability assay (MTT assay), Colony formation assay, Wound healing assay, Transwell assay, TUNEL staining and Western blotting experiments were performed, and the results proved miR-6839-5p can inhibit chondrosarcoma cells proliferation, migration and invasion. Meanwhile, miR-6839-5p significantly down-regulated apoptosis facilitator Bcl-2 expression, and promoted apoptosis of chondrosarcoma cells. It is reasonable to speculate miR-6839-5p might downregulate Bcl-2 expression to induce apoptosis in SW1353 human chondrosarcoma cells. Lastly, RNA extraction and bioinformatic analysis was performed on SW1353 cells transfected with hsa-miR-6839-5p specific mimic to investigate the potential target genes of miR-6839-5p. A total of 253 differentially expressed mRNA genes (105 up-regulated genes and 148 down-regulated genes) were found, and 23 differentially expressed downregulated genes were identified. Quantitative real-time polymerase chain reaction (qRT-PCR) was conducted to validate the results, which demonstrated the expression of BST2, VEGFA, FPR3 and PPARA was significantly downregulated by miR-6839-5p mimic. Furthermore, miR-6839-5p inhibitor can restore or partially restore the expression value of the above four genes. The analysis results of miRNA target gene prediction database indicated VEGFA was the most likely direct target gene of miR-6839-5p.

## Introduction

Chondrosarcoma is the second most common bone malignancy in adults, and surgical resection is the preferred treatment option. Due to it is often resistant to traditional chemotherapy and radiation therapy, the patients who are not suitable for surgical operation urgently needed novel therapeutic approaches. The miRNA is a non-coding single-stranded RNA of about 21–23 nucleotides in length and is essential for gene expression and cellular activity [1]. Most miRNA inhibit translation or degradation of messenger RNAs (mRNAs) by complementary binding to the genes, showing negative regulation function at the post-transcriptional level. Substantial evidence indicates that miRNA participates in various biological processes including cell growth, differentiation and apoptosis [2, 3], and miRNA dysregulation leads to the development of cancer and other diseases [4].

The role of miRNA in chondrosarcoma has been evaluated [5–10]. Some miRNAs, including miR-518b, miR-30a, miR-145, miR-125b, miR-181a, miR-126, miR-199a and miR-519d, play important roles in the development of chondrosarcoma [6, 11–19].

In previous studies (detailed in our previous paper), the expression of miR-6839-5p was significantly increased in SW1353 cells after ^125^I seed 6 Gy irradiation, which indicated miR-6839-5p may play a tumor suppression function in chondrosarcoma cells [20]. At present, the effects of miR-6839-5p on the human chondrosarcoma cells are not reported.

In this study, we valued the effects of miR-6839-5p on chondrosarcoma cells proliferation, migration, invasion and apoptosis. In addition, we conducted analysis of miRNAs–mRNAs regulatory networks, which could improve our understanding the mechanism of miR-6839-5p inhibit chondrosarcoma cells progression and provide the theoretical basis for the novel therapeutic approaches.

## Materials and methods

### Cell transfection

The human chondrosarcoma SW1353 and CAL78 cell lines were purchased from Zi Shi Biotech Co, Ltd (Shanghai, China). The cells were cultured in Dulbecco modified Eagle medium supplemented with 10% fetal bovine serum (Pan Biotech, Aidenbach, Germany), 100 U/mL penicillin, and 0.1 mg/mL streptomycin. A humidified incubator contained 5% CO_2_ was maintained at 37 ℃. For the extraction of RNA, the cells were seeded in 3.5 cm culture plates and grown to 85% confluency.

The hsa-miR-6839-5p specific mimic, mimic NC, inhibitor, and inhibitor NC (Invitrogen, USA) were configured according to the operating instructions, and the samples were mixed with diluted Lipofectamine 2000 solution (within 25 min). Subsequently, the mixture was added into the cell culture plate wells which has seeded with chondrosarcoma cells, and the plates were placed in the cell incubator for 4–6 h. Finally, discarded the cell culture medium and 500 μl DMEM high glucose medium containing 10% FBS was added into the wells, and the cells were incubated for another 24–96 h.

### Experiments on cell proliferation, migration, invasion and apoptosis

Human chondrosarcoma cells (SW1353 and CAL78) were divided into experimental groups (transfected with hsa-miR-6839-5p specific mimic) and control groups (transfected with hsa-miR-6839-5p specific mimic NC). Cell viability assay (MTT assay), Colony formation assay, Wound healing assay, Transwell assay, TUNEL staining and Western blotting experiments were performed to evaluate the effects of miR-6839-5p on chondrosarcoma cells proliferation, migration, invasion and apoptosis.

### RNA extraction and bioinformatic analysis

Due to the conditional restriction, RNA extraction and bioinformatic analysis was performed only on SW1353 cell line. The experimental and control group cells were counted and adjusted for cell concentration to 1.25 × 10^5^ cells/ml, seeded in 6-well plates (2 ml per well) and cultured overnight in the incubator. The RNA samples were obtained and purified using the MagMAX mirVana Total RNA Isolation Kit (Thermo Fisher Scientific, Waltham, Massachusetts). Nano Drop 2000 Spectrophotometer (Thermo Fisher Scientific) was applied to measure the purity and concentration of RNA samples at the absorbance of 260 nm. RNA integrity was measured by Agilent 2100 (Oebiotech, Shanghai, China). The computer sequencing was completed after library construction and library quality control. Totally, 2411 new genes were found and in which 1865 genes were functionally annotated.

### Real-time quantitative PCR

Quantitative real-time polymerase chain reaction (qRT-PCR) was conducted to validate the results of the miRNAs microarray analysis, along with the predicted target genes. The qRT-PCR reactions were performed according to the Applied Biosystem 7500 Real-Time PCR System kit instructions from TaKaRa Corporation. Set the instrument reaction conditions: 95 ℃ (30 s), 95 ℃ (5 s), 60 ℃ (34 s), a total of 40 cycles. The primers are given in Table 1.

**Table 1:** Primer sequences for Real-time PCR

miRNAs	Forward	Reverse
U6	GTAACCACCTTGGTGTCCTTGTCC	GGCCAACCGCGAGAAGATGTTTTTTTTT
hsa-miR-6839-5p	AGTCTGGATTGAAGAGACGACCCA	GGCCAACCGCGAGAAGATGTTTTTTTTT
GAPDh	GGAGCGAGATCCCTCCAAAAT	GGCTGTTGTCATACTTCTCATGG
NAIP	GAGGGTGAAGCTGGAAGTGGAAAG	TCGTCTGGTCTGGTGGAACTAAGG
PROM1	GTGGCGTGTGCGGCTATGAC	CCAACTCCAACCATGAGGAAGACG
BST2	CGCCACCTGCAACCACACTG	TCTCCCTCAAGCTCCTCCACTTTC
VEGFA	GCCTTGCCTTGCTGCTCTACC	GGTCTCGATTGGATGGCAGTAGC
FPR3	TTCAGCGTGCCTATGTCCATCATC	GCCACCACAGCAGCGAAGAC
PPARA	TCGGCGAGGATAGTTCTGGAAGC	ACCACAGGATAAGTCACCGAGGAG
RAB5B	GGGTGCCCAAGCTGCAATCG	GCCTGTCGCTGTAGTTCCTTCAC
PPP1R12B	ACAAGCCAGAAGAGCCCAAAGATG	GAGCCTCGGTCCCTTATAGGTTCC

### Statistical analysis

All experiments were repeated three times. Statistical analysis performed with Student’s t-test and one-way analysis of variance (ANOVA) methods using SPSS 16.0 and Graph Pad Prism 5 software. The results were expressed as $\overline{x}$ ± S. Statistical significance was determined as p < 0.05.

## Results

### miR-6839-5p inhibits the proliferation of human chondrosarcoma cells

**Fig. 1:**
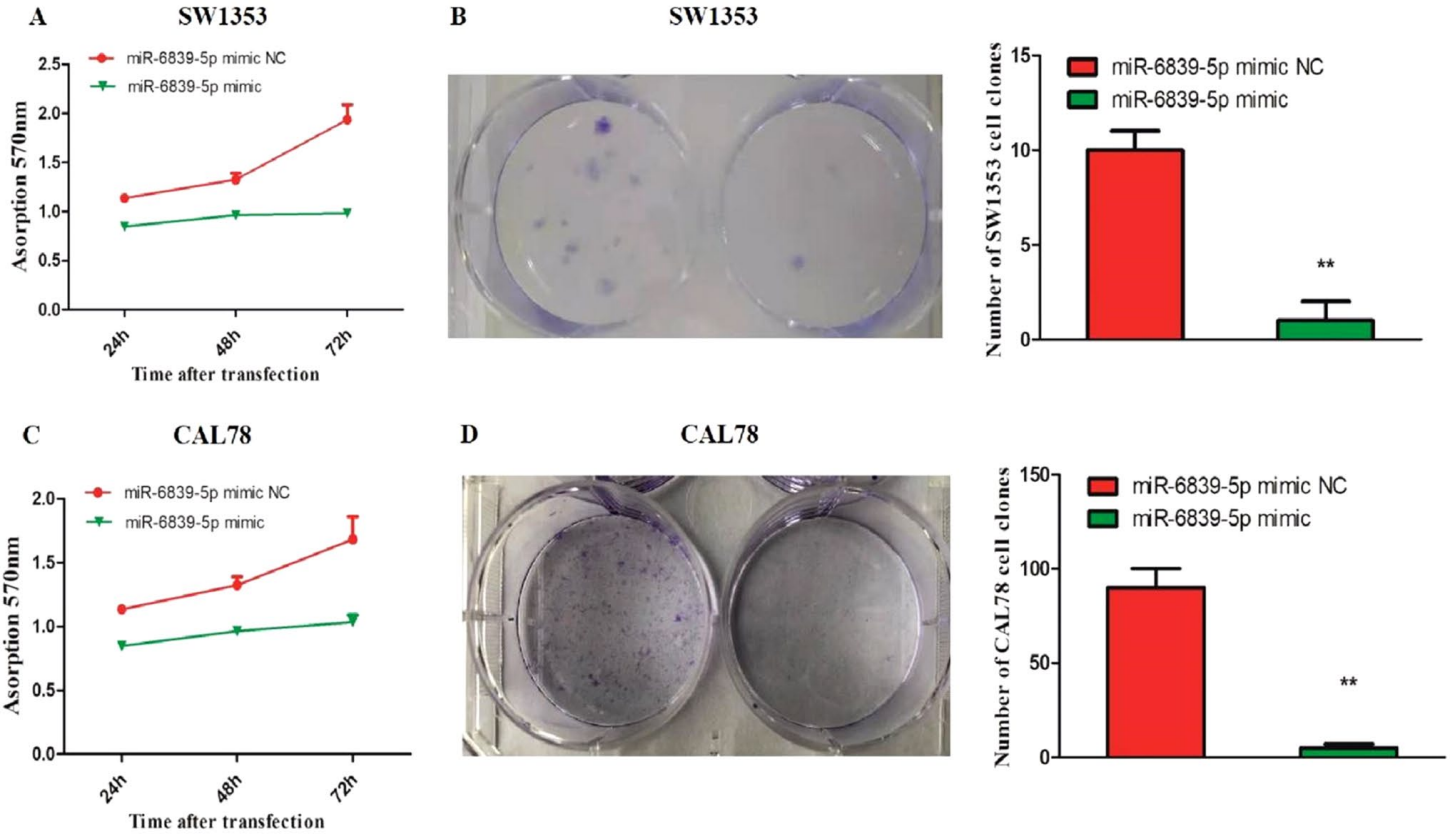
Growth inhibitory effect of miR-6839-5p in SW1353 and CAL78 cells. **A**. Cell proliferation of SW1353 cells transfected with miR-6839-5p mimic was significantly reduced 24 h after the transfection. **B**. Clone forming capacity of SW1353 cells transfected with miR-6839-5p mimic was as 10% as the control cells’ 24 h after the transfection. **C**. Cell proliferation of CAL78 cells transfected with miR-6839-5p mimic was significantly reduced 24 h after the transfection. **D**. Clone forming capacity of CAL78 cells transfected with miR-6839-5p mimic was as 5.56% as the control cells’ 24 h after the transfection

We detected miR-6839-5p’s effect on the proliferation of human chondrosarcoma cells using the MTT assay and Colony formation assay. As shown in Fig. 1, the experimental group cells transfected with miR-6839-5p mimic showed significantly lower cell proliferation rate compared with the control group cells transfected with mimic NC (P < 0.05). At 24 h after the transfection, SW1353 experimental group cells’ clone formation capacity was as 10% as the control group cells’, and CAL78 experimental group cells’ clone formation capacity was as 5.56% as the control group cells.

### miR-6839-5p inhibits the migration of human chondrosarcoma cells

**Fig. 2:**
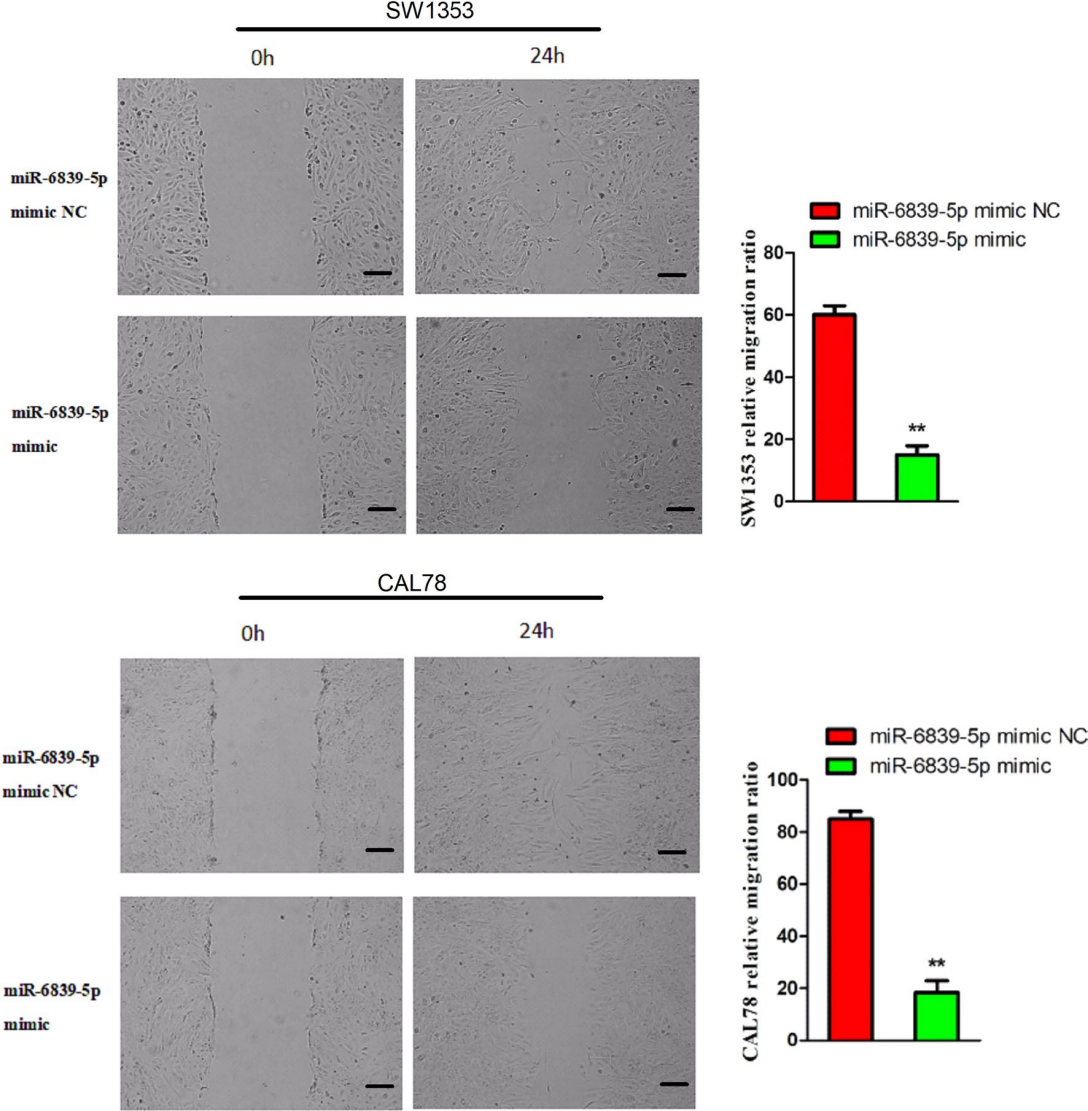
Migration inhibition of SW1353 and CAL 78 cells by miR-6839-5p (40 ×). Cell mobility was significantly reduced by the transfection with miR-6839-5p mimic. The scale bar is 100 μm. The migration rate of SW1353 control cells was 60%, and experimental group cells migration rate was 15%. The migration rate of the CAL 78 cells was 82% in the control group and 23% in the experimental group

To investigate the effect of miR-6839-5p on chondrosarcoma cells migration, we examined the migration of SW1353 and CAL78 cells by wound healing assay. As shown in Fig. 2, the experimental group cells showed significantly lower migration rate compared with the control group cells (P < 0.05). In the experimental group, SW1353 cells migrated 15% and CAL78 cells migrated 23% 24 h after the scratch. On the contrary, SW1353 control group cells migrated 60% and CAL78 control group cells migrated 82%.

### miR-6839-5p inhibits the invasion of human chondrosarcoma cells

**Fig. 3:**
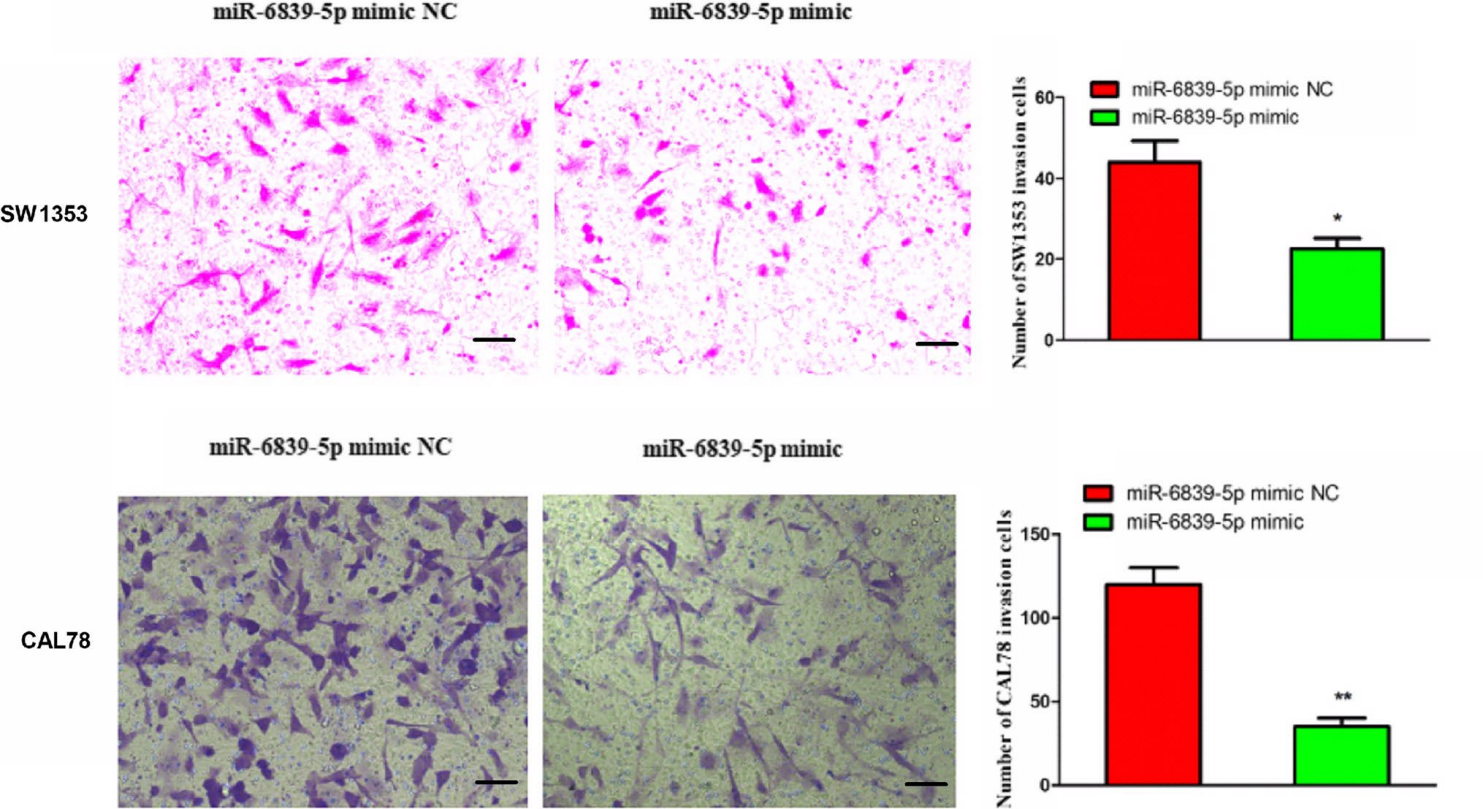
The invasion inhibition effect of miR-6839-5p on SW1353 and CAL 78 cells (100 ×). In the experimental group cells transfected with miR-6839-5p mimic, the number of cells crossing the Transwell compartment basement membrane coated with Matrigel matrix gel decreased, with a significant decrease in invasiveness. The scale bar is 200 μm. Compared to the control group cells, the experimental group cells’ invasion rate of SW1353 and CAL78 was 51.52% and 29.17%, respectively

The effect of miR-6839-5p on chondrosarcoma cells invasion was evaluated by Transwell assay. As shown in Fig. 3, the amount of experimental group cells crossed the basement membrane of the Transwell compartment was obviously less than the control group cells (P < 0.05). Compared to the control group cells, SW1353 and CAL78 experimental group cells’ invasion rate was 51.52 and 29.17%, respectively.

### miR-6839-5p promotes apoptosis in human chondrosarcoma cells

**Fig. 4:**
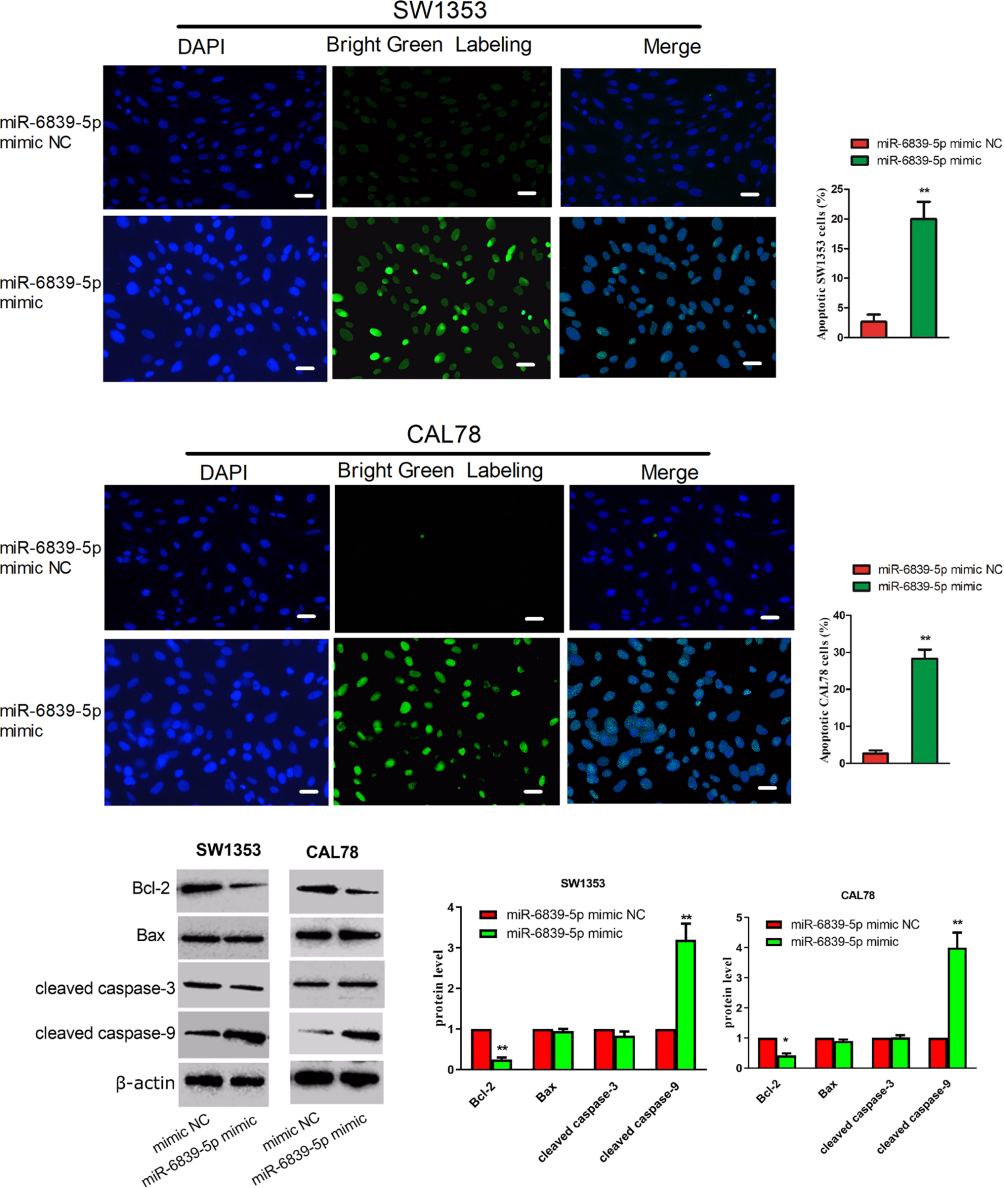
Pro-apoptotic effect of miR-6839-5p on SW1353 and CAL 78 cells (400 ×). The experimental group of cells transfected with miR-6839-5p mimic underwent apoptosis in situ. The blue fluorescent staining part is the nucleus, and the green fluorescent staining part inside the cell is the broken DNA. The scale bar is 300 μm. SW1353 cells apoptosis rate was 2.67% in control group, and 20.0% in the experimental group. CAL78 cells apoptosis rate was 22.33% in control group, and 51.07% in the experimental group. Compared with the control group cells, SW1353 and CAL78 cells transfected with miR-6839-5p mimic showed significantly decreased expression of Bcl-2 and increased expression of cleaved caspase-9.The fold change of the expression level of Bcl-2 after the treatment with miR-6839-5p mimic in SW1353 and CAL 78 cells was 0.25 and 0.42, respectively. The fold change of the expression level of cleaved caspase-9 after the treatment with miR-6839-5p mimic in SW1353 and CAL 78 cells was 3.2 and 4.0, respectively

To investigate the effect of miR-6839-5p on apoptosis in chondrosarcoma cells, we examined experimental and control group cells in situ apoptosis by TUNEL staining assay. The results are shown in Fig. 4, compared with the control group cells, the experimental group cells underwent apoptosis, showing abnormal manifestations of nuclear fragmentation, cavity formation, and DNA breakage. SW1353 cells apoptosis rate was 2.67% in control group, and that was 20.0% in experimental group. CAL78 cells apoptosis rate was 22.33% in control group, and that was 51.07% in experimental group.

To further validate the effect of miR-6839-5p on apoptosis, we examined the expression of Bcl-2, Bax, cleaved caspase-3, and cleaved caspase-9 apoptotic proteins in chondrosarcoma cells by Western blotting assay. Compared to the control group cells, SW1353 and CAL78 transfected with miR-6839-5p mimic showed significantly decreased expression of Bcl-2 and increased expression of cleaved caspase-9. The fold change of the expression level of Bcl-2 after the treatment with miR-6839-5p mimic in SW1353 and CAL 78 cells was 0.25 and 0.42, respectively. The fold change of the expression level of cleaved caspase-9 after the treatment with miR-6839-5p mimic in SW1353 and CAL 78 cells was 3.2 and 4.0, respectively.

Bax and cleaved caspase-3 expression in the two group cells was not obviously different, and the same trend was observed in the two cell lines.

### miR-6839-5p affect mRNA expression in human chondrosarcoma SW1353 cells

**Fig. 5:**
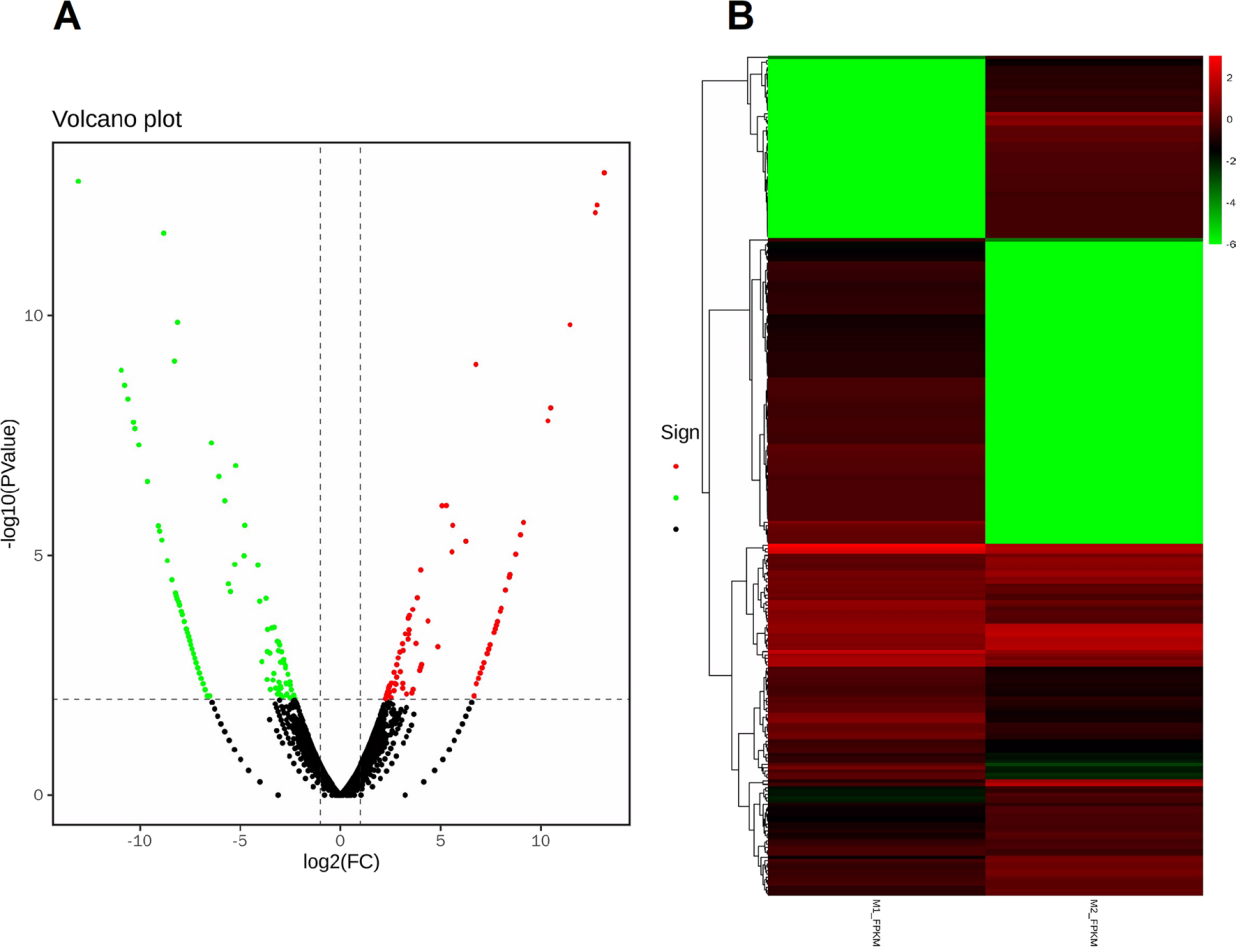
Differentially expressed genes in SW1353 cells (M2) transfected with miR-6839-5p mimic and in the control group cells (M1) transfected with miR-6839-5p mimic NC. **A**. The volcano plot. Every dot in the plot represents a gene in M_2_, black represents non-differentially expressed genes compared with M1, red dots represent up-regulated differentially expressed genes, and green dots represent downregulated differentially expressed genes. The abscissa represents the log value of the fold difference of a gene expression in the two groups, and the greater the absolute value, the greater the fold of expression difference between the two groups. The ordinate represents the negative log value of the P value, with larger ordinate indicating more significant differential expression. **B**. Rows in the plots represent individual gene, and columns represent individual sample. The abscissa are the sample name and their clustering results, and the ordinate are the differentially expressed genes and their clustering results. Colors represent the expression level of every gene in the sample, as indicated by log10

Edge R software was used to screen the difference between SW1353 cells (M1) transfected with miR-6839-5p mimic NC and experimental cells (M2) transfected with miR-6839-5p mimic [21]. The difference multiple (Fold Change) greater than or equal to 2 and p-value less than 0.01 were used as the screening criteria. Transcriptome analysis identified 253 differentially expressed genes, including 105 up-regulated genes and 148 down-regulated genes (Fig. 5A). The cluster histogram shows the different gene expression profiles between M1 and M2 group, and the heatmap shows the genes differentially expressed between the two groups (Fig. 5B). In order to find the target genes regulated by miR-6839-5p, we crossed the 148 downregulated differentially expressed genes identified by the transcriptome screen with the results predicted by TargetScan, miRWalk or miRDB, and we found 23 differentially expressed downregulated target genes (Table 2).

**Table 2:** 23 downregulated target genes differentially expressed between M2 and M1

mRNAs	Average expression in M1 group	Average expression in M2 group	Fold change	ANOVA (*p*-value)
NAIP	0.766216	0	− 10.60272296	5.63E-09
RBFOX1	0.118533	0	− 7.267779292	0.001391155
KCNQ2	0.043212	0	− 7.116816014	0.002220049
ADARB2	0.037184	0	− 6.855858991	0.00481584
PhYhIPL	0.075012	0	− 6.651323048	0.008564159
PROM1	0.026806	0	− 6.651323048	0.008564159
hhIPL1	0.035915	0	− 6.537041374	0.008564159
ALDh8A1	0.10455	0	− 6.537041374	0.008564159
BST2	6.569082	0.073125	− 6.448535346	4.58E-08
IFI44L	1.269392	0.008372	− 6.056113014	2.30E-07
PMF1-BGLAP	4.276152	0.054897	− 5.768906224	7.29E-07
MGAT4A	0.257618	0.011575	− 3.638282012	0.001012527
SLC26A10	0.407108	0.033049	− 3.323600668	0.002910357
OAS2	8.654291	0.937226	− 3.302786555	0.000314625
FPR3	0.26936	0.018576	− 3.234611852	0.005932006
ARL17A	3.022951	1.133049	− 3.151672949	0.000626352
PPARA	15.366781	6.18231	− 2.942859882	0.001024817
GVQW3	0.605723	0.298099	− 2.919753752	0.001684191
ZNF260	4.032847	0.621106	− 2.74382718	0.002225252
RAB5B	29.526409	6.506531	− 2.741061575	0.00203173
ACSBG1	0.469566	0.069749	− 2.714345704	0.004595519
IL10RA	0.505598	0.078613	− 2.665306194	0.007791452
PPP1R12B	2.700869	1.466434	− 2.324697972	0.008254139

### Fluorescence quantitative real-time PCR analysis of the differentially expressed target genes

To investigate the potential target genes of miR-6839-5p in chondrosarcoma SW1353 cells, we performed transcriptome sequencing and analysis in the control group cells (M1) transfected with miR-6839-5p mimic NC and experimental group cells (M2) transfected with miR-6839-5p mimic.

**Fig. 6:**
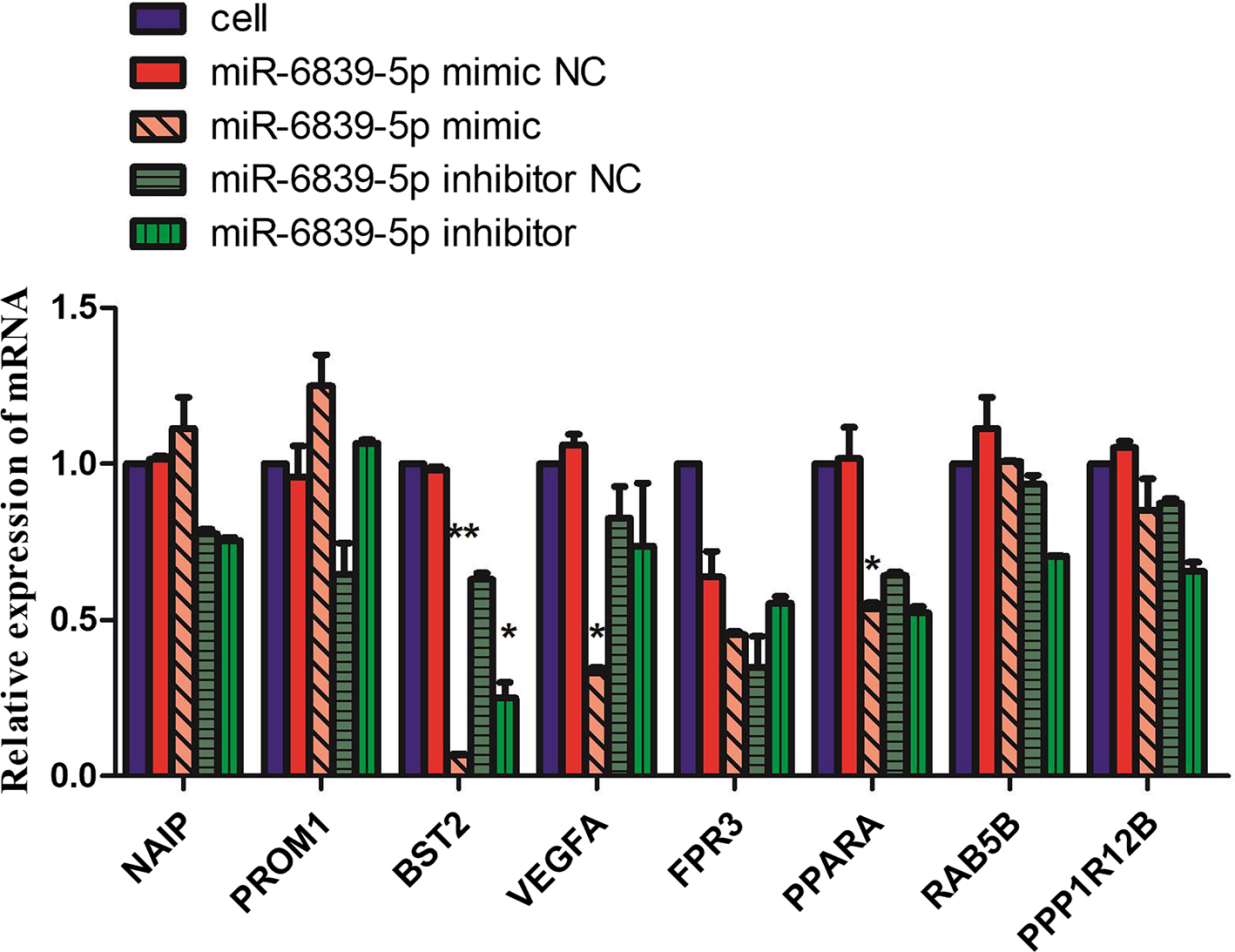
qRT-PCR analysis of the eight differentially expressed genes between the experimental and control groups. *represent mRNA expression fold change of target genes was < 0.5 compared to control, **represent mRNA expression fold change was < 0.1 compared to control

We searched potential target genes of miR-6839-5p using miRNA databases (TargetScan, miRWalk and miRDB). We query the https://pubmed.ncbi.nlm.nih.gov/ website for relevant information about the 23 differentially expressed genes in Table 2. Then, we selected eight possible target genes of miR-6839-5p in SW1353 cells and performed qRT-PCR experimental validation of the eight predicted target genes described above. The results demonstrated that the expression of BST2, VEGFA, FPR3 and PPARA was down-regulated significantly in M2, while miR-6839-5p inhibitor can restore or partially restore the expression value of the above four genes in SW1353 cells. (Fig. 6).

## Discussion

The miRNA is a class of tiny non-coding RNA that regulates gene expression at the transcriptional and post-transcriptional levels by binding to specific sequences of target genes, and is an important regulator in physiological processes and pathological conditions. Dysregulation of miRNA can cause a variety of tumors and also plays an important role in the development of chondrosarcoma [4, 11, 22].

In Previous studies (detailed in our previous paper), the expression of miR-6839-5p was significantly increased in SW1353 cells after ^125^I seed 6 Gy irradiation, which indicated miR-6839-5p may play a tumor suppression function in chondrosarcoma cells [20]. However, the effects of miR-6839-5p on chondrosarcoma cells have not been reported.

Based on the results of this study, miR-6839-5p showed anti-proliferation, anti-migration, and anti-invasive effects in both SW1353 and CAL78 cell lines. MiR-6839-5p displayed promoting higher apoptosis in the cells and causing the down-regulation of Bcl-2 expression. There are two distinct pathways causing apoptosis, namely the intrinsic and extrinsic pathway [23]. In which the endogenous pathway is tightly regulated by the Bcl-2 family member [24]. Proteins from the BCL-2 family control cell survival and apoptosis in health and disease, and regulate apoptosis-unrelated cellular processes. In this study, Bcl-2 expression was significantly downregulated in the experimental group transfected with miR-6839-5p mimic. Therefore, it is reasonable to speculate miR-6839-5p might downregulate Bcl-2 expression to induce apoptosis in SW1353 human chondrosarcoma cells through the endogenous pathway.

To further identify the target genes regulated by miR-6839-5p, we crossed the 148 downregulated differentially expressed genes identified by transcriptome screening with the results predicted by TargetScan, miRWalk and miRDB. We found 23 differentially expressed downregulated target genes, and selected 8 of them for qRT-PCR validation. The results showed the expression of BST2, VEGFA, FPR3 and PPARA was significantly downregulated in SW1353 cells transfected with miR-6839-5p mimic, which indicated miR-6839-5p may inhibit the proliferation, migration, invasion and promote apoptosis by regulating BST2, VEGFA, FPR3 and PPARA. Furthermore, The analysis results of miRNA target gene prediction database indicated VEGFA was the most likely direct target gene of miR-6839-5p.

Angiogenesis is critical to the growth, invasion and metastatic properties of chondrosarcoma [25–27]. VEGFA is an important factor of angiogenesis during tumor growth [28–33]. Its expression is regulated by leptin, adiponectin, CCL 5 and both terminal regulatory proteins. VEGFA upregulated in chondrosarcoma cell lines and is associated with chondrosarcoma grade [18, 34–36]. Although chondrosarcoma staging increases VEGFA, it is difficult to use it as a biomarker because angiogenesis always occurs with the growth of the tumor and not with the malignant behavior. Antiangiogenic therapy has been proposed for treatment of high grade chondrosarcoma based on the evidence of increased microvessel density and intracartilaginous vascularity in chondrosarcoma [37, 38].

We performed transcriptome analysis and validation experiments of SW1353 cells transfected with miR-6839-5p mimic NC and mimic, which showed VEGFA expression was decreased significantly in cells transfected with miR-6839-5p mimic. Meanwhile, miR-6839-5p inhibitor restored or partially restored the expression of the gene, which indicated VEGFA may be a target gene of miR-6839-5p. The relationship between miR-6839-5p and VEGFA will be further verified by dual luciferase reporter assay and by co-transfection of miR-6839-5p mimic and VEGFA plasmids in chondrosarcoma cells.

Our study identified an important role of miR-6839-5p in regulating chondrosarcoma cells growth, and miR-6839-5p may be a potential therapeutic target for human chondrosarcoma in the future.

## Limitations

In this study, we selected 8 predicted target mRNA genes of miR-6839-5p for verification. However, there may be other mRNAs that should be further studied in the future. In addition, the specific regulatory mechanisms of miR-6839-5p require more attention in future studies. Lastly, due to the complex regulatory network of miRNA on target genes and the limited genes we tested in the experiment, it is not possible to determine which target genes miR-6839-5p acts through based on our experimental results. Additional studies are necessary to further uncover the relationship between miR-6839-5p and VEGFA in chondrosarcoma cells.

In order to reduce the workload and save the cost, we cut the PVDF membrane according to the molecular weight Marker, and the cut membrane was closed and incubated with antibody, respectively. Due to the development techniques and other reasons, our stips did not show the membrane edges.

## Conclusion

In summary, our study demonstrated miR-6839-5p can inhibit chondrosarcoma cells proliferation, migration and invasion. Next, miR-6839-5p downregulated Bcl-2 expression and induce apoptosis in human chondrosarcoma cells. In addition, VEGFA was the most likely direct target gene of miR-6839-5p. Our findings presented here provide insight into the effects of miR-6839-5p on chondrosarcoma cells, along with the theoretical basis for the novel therapeutic approaches.

## Data Availability

The data used and/or analyzed during the present study are available from the corresponding author on reasonable request.
